# Quality of Life and Cost-Effectiveness of Radiofrequency Ablation versus Open Surgery for Benign Thyroid Nodules: a retrospective cohort study

**DOI:** 10.1038/srep37838

**Published:** 2016-11-24

**Authors:** Wen-Wen Yue, Xiao-Long Li, Hui-Xiong Xu, Feng Lu, Li-Ping Sun, Le-Hang Guo, Ya-Ping He, Dan Wang, Zhi-Qiang Yin

**Affiliations:** 1 Department of Medical Ultrasound, Shanghai Tenth People’s Hospital, Ultrasound Research and Education Institute, Tongji University School of Medicine, Shanghai, 200072, China; 2 Thyroid Institute, Tongji University School of Medicine, Shanghai, 200072, China; 3 Shanghai Center for Thyroid Diseases, Shanghai, 200072, China; 4 Center of Diagnosis and Treatment for Thyroid Diseases, Shanghai Tenth People’s Hospital, Tongji University School of Medicine, Shanghai, 200072, China

## Abstract

This study is to compare the health-related quality of life (HRQoL) and cost-effectiveness of radiofrequency ablation (RFA) and open thyroidectomy (OT) for benign thyroid nodules (BTNs) treatment. HRQoL and utility were assessed for 404 BTN patients immediately before treatments (RFA:OT = 137:267) and at 6-month visit. A cost-effectiveness analysis was performed from societal perspective in the China context. Resource use (hospitalization, sick leaves) was collected. We used the net monetary benefit approach and computed cost-effectiveness acceptability curves for RFA and OT. Sensitivity analyses of costs of RFA were performed. At 6-month visit, patients treated with RFA had significantly better HRQoL than patients treated with OT on general health (68.5 versus 66.7, *P* = 0.029), vitality (71.3 versus 67.5, *P* < 0.001) and mental health (80.9 versus 79.3, *P* = 0.038). RFA was more effective than OT in terms of quality-adjusted life-years (QALYs; 0.01QALY/patient) but more expensive (US$823/patient). The probability that RFA would be cost effective at a US$50,000/QALY threshold was 15.5% in China, and it would be increased to 88.4% when price of the RFA device was lowered by 30%. RFA exhibited a significant improvement of HRQoL relative to OT, but is unlikely to be cost effective at its current price in short time.

Although surgical resection is traditionally the well-established and mainstay of treatment for benign thyroid nodules (BTNs)^1^,^2^, the following 2–10% risk of complications such as bleeding, post-operation infection and permanent recurrent laryngeal nerve palsy are still not insignificant^3^. Moreover, patients following thyroid surgery have a high frequency of hypothyroidism and neck scar complaints, which adversely affects the health-related quality of life (HRQoL)^4^. To reduce these side effects, recently various nonsurgical minimally invasive techniques have been introduced—radiofrequency ablation (RFA) being just one of them^5^. RFA has become widespread in clinical practice and has both extended the range of treatment options but confused the correct selection. Several recently published studies comparing RFA with conventional open thyroidectomy (OT) in select BTN patient populations have shown clinical superiority of RFA over OT^6^,^7^, mainly for its inherent advantages in quicker recovery, fewer complications and shorter hospital stays. While effectively superior, towards HRQoL whether the optimally medical therapy for BTNs is RFA or OT remains uncertain.

HRQoL can provide a subjective assessment of the impact of treatment across psychological, physical, social and somatic status^8^, and the comprehensive predictions of the effect of therapy will benefit clinicians as well as patients under specific conditions. More importantly, there is agreement that HRQoL is a central aim of the medical treatment, and methods of its evaluation are under continuous scrutiny^8^. Although currently HRQoL data are not needed in innovative technology development, they are increasingly used as part of the evidence submitted for drug approval^9^. Therefore, this comparative study of RFA and OT on HRQoL will be of great realistic significance. Moreover, considering RFA is a cost endeavor, its routine use should better be supported by a well conducted cost-effectiveness analysis to balance against its impact on HRQoL and costs. Also, as life expectancy of the patients with BTNs is generally not shortened by the disease, quality adjusted life year (QALY) evaluations are particularly relevant. The current literature is lacking rigorous data, and utility values between RFA and OT have been rarely assessed. The purpose of this study was to present the HRQoL outcomes and a cost effectiveness evaluation among patients with BTNs that treated with RFA relative to those who received OT, also to compare the RFA and OT results of HRQoL against data from the general population.

## Patients and Methods

This retrospective study was approved by the Ethics Committee of the Shanghai Tenth People’s Hospital. The requirement to obtain informed consent was waived because of the retrospective nature of the study, but a written informed consent was obtained from each subject before RFA or OT after full explanation of the purpose and nature of the procedure used. The study was performed in accordance with relevant guidelines and regulations.

## Patients

**Figure 1:**
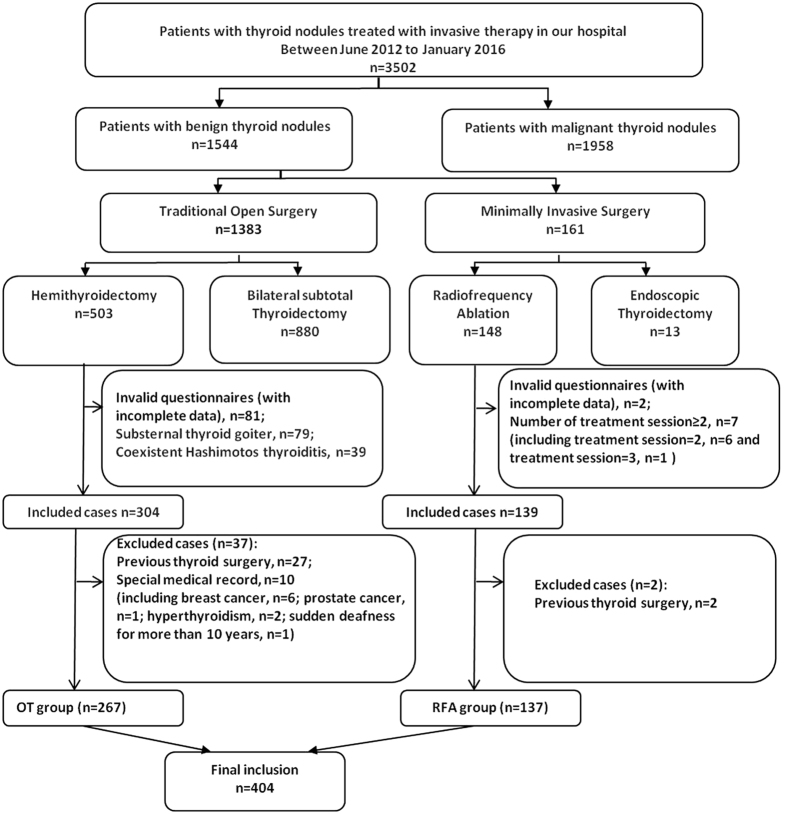
Flow chart for the management of thyroid nodules in our hospital. RFA group, patients treated with radiofrequency ablation; OT group, patients treated with open thyroidectomy.

From June 2012 to January 2016, 3502 patients underwent invasive therapy for TN(s) as a first-line treatment in our center. Among them, 404 patients were included in this study and were classified into RFA group (n = 137) or OT group (n = 267) according to the initial treatment strategy (Fig. 1). In our institution, RFA or OT was considered if a patient with TN(s) reported of compressive symptoms or cosmetic problems or anxiety about a malignancy. For RFA there was an additional requirement that cytologic confirmation of benign nature of the nodule with ultrasound (US)-guided fine needle aspiration cytology (FNAC) examination according to the American Bethesda System for Reporting Thyroid Cytopathology^10^ and nodule without changes on US at least 12-month. Also, for the patient ineligibility to undergo surgery for high thyroid surgical risk (poor surgical candidates, falling general anaesthesia due to a medical condition, repeated neck dissection), RFA would be required. Further, for a patient that was suitable for both RFA and OT, the definitive treatment modality was “self-selecting” after a full explanation of the differences between two procedures. The RFA patients were diagnosed cytologically and the OT patients were diagnosed by surgical pathology. For patients in the RFA group, 48.9% (67/137) had clinical symptoms or cosmetic problems and the rest of them were anxious about a malignant change; while for patients in the OT group, 36.7% (98/267) were been diagnosed with thyroid nodules that assessed as suspicious for malignancy according to the US Thyroid Imaging Reporting and Data Systems (TI-RADS) (rated ≥ 4)^11^ following the regular medical checkup, 32.6% (87/267) had clinical symptoms, 22.1% (59/267) with nodules increased obviously in a short time (double in size within 6 months) and 8.6% (23/267) were anxious about a malignant change. All the enrolled patients fulfilled the following criteria: patients with valid questionnaires (with complete data); underwent a single treatment method of RFA or hemithyroidectomy; serum levels of thyrotropin, thyroid hormone and calcitonin within normal limits. Exclusion criteria included patients who had already treated with contralateral thyroid lobectomy. Further, patients with major comorbidities that suspected to have a substantial HRQoL impact (e.g. malignant tumors, chronic obstructive pulmonary disease(COPD), congestive heart failure)^12^ were also excluded.

## General population samples

HRQoL data from the general population were derived from a survey which had the same study area (Shanghai, China) as in our study and conducted by Wang R *et al*. using Mandarin version of Short Form-36(SF-36) in 2008^13^,^14^. The survey was based on a region-stratified random sample of adult Shanghai, China citizens (18 years or older) using a self-finished interview method. The respondents filled in the self-administered questionnaires in their home or in local resident comittees. The questionnaire included general information (ie, sociodemographic variables, comorbidity and medication) and the tested Mandarin version of SF-36. A total of 1024 subjects actually completed the questionnaire. This study also tested the reliability of the Mandarin version of SF-36 by randomly selecting 10% of the total number of the respondents to refill the questionnaires 2–7 days after the baseline test.

## Treatment of BTNs and Follow-up

Conventional US, contrast enhanced ultrasound (CEUS), US-guided FNAC and RFA were carried out using a ML6–15 liner transducer (frequency range: 6–15 MHz), a real-time US system (GE LogiqE9, GE Healthcare, WI, USA) and a bipolar RFA system. The bipolar RFA instrument (Celon AG Medical Instruments, Teltow, Germany) consists of an RF generator with a frequency of 470 KHz and an maximum power output of 250 W, designed technically overcoming the disadvantages of use of grounding pads. The bipolar RFA electrode used in this study was 15.5-gauge and 15-cm long with a conducting part of 20 mm in length (150-T20). For 150-T20, an internally cooled electrode was provided with a peristaltic pump perfusing 0.9% NaCl solution at 30 mL/min.

In RFA group, all the procedures were conducted by a radiologist with 5 years of experience in thyroid RFA using the previously described standard RFA techniques such as “hydrodissection technique” and “moving-shot technique”^5^,^6^,^7^. The ablation procedures were monitored by real-time US, and ablations were not terminated until the transient hyperechoic cloud caused by the gas covered all units of the nodule. For patients with multiple nodules, only the largest nodule was subject to treatment and those patients formed the RFA group. All patients were closely observed for 20–30 min after RFA treatments. For OT patients, all the operations were carried out by general surgeons with 6 years’ clinical experience under general anesthesia according to the standard operation method of hemithyroidectomy as suggested in previous studies^6^,^15^. Postoperative pain and serum calcium were assessed 24 hours after surgery.

US assessments including gray scale US and color Doppler US were performed for the RFA group patients at 1, 3, 6 and 12 months after treatment and every 6 months thereafter. Thyroid function tests were performed one month after treatment for patients of the two groups, and if any functional anomalies were demonstrated, they needed to be evaluated every month until normalization. For the hypothyroid patient, thyroid function was examined to adjust the optimal dosage of levothyroxine (Euthyrox) every month thereafter. For the euthyroid patients the tests were reassessed at the 6-month follow-up. Any adverse event that occurred immediately after treatment and during follow-up period was also addressed.

## HRQoL and Utility

Quality of life (QoL) was assessed using the tested self-administered Mandarin version of SF-36 immediately before RFA or OT and at 6 months visit. The SF-36 questionnaire was used to describe the QoL because it is the most extensively validated and used instrument to measure generic HRQoL and has been shown to be sensitive in patients with benign and malignant thyroid nodules^16^. The questionnaires were filled in by the patients themselves on the spot when patients came to the hospital or by mail. And one mailed reminder or a phone call was sent after ten days to non-responders. The assessment scores that generated across eight dimensions of health (physical functioning (PF), role-physical (RP), bodily pain (BP), general health (GH), vitality (VT), social functioning (SF), role-emotional (RE), mental health (MH)) were calculated by a standard scoring algorithm of the responses across scale items with higher scores indicating better health. Clinical data (ie, comorbidities, previous operation history, specific diagnosis, smoking and alcohol consumption) and demographic parameters (ie. sex, age, ethnic group, marital status, current job) were obtained by medical records review.

The EuroQoL five-dimensions(EQ-5D-3L) self-report questionnaire which has been recommended as a tool for conducting health technology assessment in China and whose descriptive system has been validated in Chinese populations^17^,^18^, was used to assess utility and QALYs before treatment and at the 6-month visit. At each assessment, the utility value was acquired through a utility function, which was calculated on the base of the revealed preferences of the Chinese population^19^. For each patient, QALY was evaluated by weighting the time period between two assessments (6-month in this study) with the established utility value, supposing a linear change in utility over time (ie, using a formula of trapezoidal area).

## Resource Use and Costs

Resource use was collected from the admission for BTNs until the patient assessment at 6 months after treatment. Cost identification consisted of direct cost and indirect cost. Direct cost included the hospitalization for RFA, OT, as well as any consumption cost that related the adverse event induced by the procedure. Levothyroxine (Euthyrox) was used for 8.6% patients (23/267) in the OT group during the visit at 6 months. The price of Euthyrox was obtained from the China drug database. The cost of hospitalization was acquired by reviewing the billing details of hospitalization expense. Since most of those in the study were Shanghai natives, transportation costs were not included in the study. Costs were assessed from the China societal perspective and were expressed in CNY (results are also presented in US dollars using the conversion rate of Y1 = US$0.1505). Unit costs data are listed in Table 1. Indirect costs were evaluated using the human capital approach according to the Chinese guidelines for cost-effectiveness studies and using a standard formula: Indirect costs = Gross Domestic Product (GDP) Per Capita*Days*Productive Weight (where productive weight is according to the internationally weighted value and that the ages can be divided into four groups: 0–14, 15–44, 45–59, >60 years old and the weighted value were 0, 0.75, 0.80, 0.10, respectively). The value of GDP Per Capita was based on the Shanghai, China regional value estimated at Y267 (US$40.2) per day.

## Cost-Effectiveness Analysis

The time horizon was fixed at 6 months and effectiveness was reported in QALYs. We used the incremental cost effectiveness ratio (ICER) as the only outcome measurement to compare RFA with OT methods with a formula of: ICER = (cost of RFA − cost of OT)/(QALYs of RFA − QALYs of OT). A strategy was regarded cost-effective over the other if the ICER was <US$50,000/QALY^20^.

## Statistical Analysis

**Figure 2:**
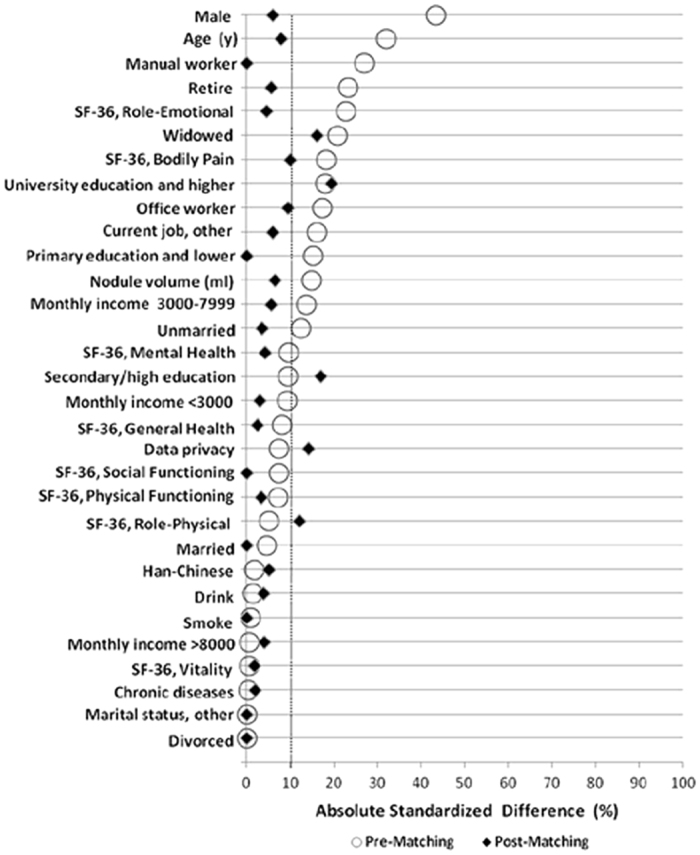
Absolute standardized differences. Absolute standardized differences comparing the baseline characteristics of patients with benign thyroid nodules treated with radiofrequency ablation or open thyroidectomy before and after propensity score matching.

Because patients were not randomized to undergo RFA versus OT, a propensity score model was used to compare the QoL of patients with BTNs that treated with RFA relative to those who received OT. The propensity score presents the conditional probability of receiving an exposure given a vector of the measured covariates^21^. In our study, propensity scores for all the patients were estimated by multiple logistic-regression models using the following baseline characteristics as covariates: sex, age, nodule volume, ethnic group, SF-36 dimension scores (PF, RP, BP, GH, VT, SF, RE, MH), smoke, drink, chronic diseases, marital status, monthly income, education lever and current job^22^. Before matching, the mean propensity score was 0.425 for patients in the RFA group (n = 137) and 0.295 for patients in the OT group (n = 267), with a standardized difference of 80.7% (t-test, *P* < 0.001). A 1:1 matched study group was created by using the nearest neighbor method (caliber = 0.05). After matching, the mean propensity score was 0.377 for patients of the RFA group (n = 108) and 0.374 for patients of the OT group (n = 108), with a standardized difference of 2% (t-test, *P* = 0.887). In our study the standardized difference (the difference in means between two groups divided by the pooled standard deviation) was used to assess the balance of all baseline covariates between two groups before and after propensity score matching (Fig. 2). A 20 percent standardized difference between two groups indicates a small difference^21^, and a 10 percent standardized difference might correspond to the smallest potentially meaningful difference^23^. Our propensity score model was discriminated effectively and well calibrated between patients who underwent RFA and OT at baseline. Before and after the 1:1 matching, continuous variables were compared by using the independent t-test or Mann-Whitney U test between the two groups. Qualitative variables were analyzed using Chi-square test or Fisher’s exact test. Differences in the mean SF-36 scale scores between patients in the two treatment groups and the general population were analyzed with the one sample t-test.

A nonparametric conditional bootstrap with 10,000 replications was performed to estimate the 95% CIs for QALYs, costs and the cost-effectiveness acceptability curves. A one-way sensitivity analysis was carried out reducing the price of RFA by 10% and 30%, because as technology developed, a highly efficient and low-cost RFA may be available in the future. Statistical analyses were performed by using the SPSS software (version18.0; SPSS, Chicago, III).The significance level was defined as *P* value of less than 0.05.

## Results

### Baseline Characteristics

Baseline clinical and demographic parameters are shown in Table 2. Due to the inevitable selection bias, compared with patients in the RFA group, those in the OT group had a higher prevalence of higher age, retired males, smaller tumor volume, lower SF-36 scale scores in GH, RE and MH. After performing propensity-score matching for the entire population, a total of 108 matched patient pairs were created and clinical characteristics or SF-36 scale scores did not differ significantly between two groups at enrollment. The RFA group consisted of 72 women (mean age, 52.0 years; age range, 18–69 years) and 36 men (mean age, 48.3 years; age range, 20–70 years). The OT group consisted of 69 women (mean age, 51.5 years; age range, 19–71 years) and 39 men (mean age, 41.9 years; age range, 19–71 years). The median nodule volume at initial was 5.6 ml in the RFA group and 5.3 ml in the OT group.

### HRQoL

**Figure 3:**
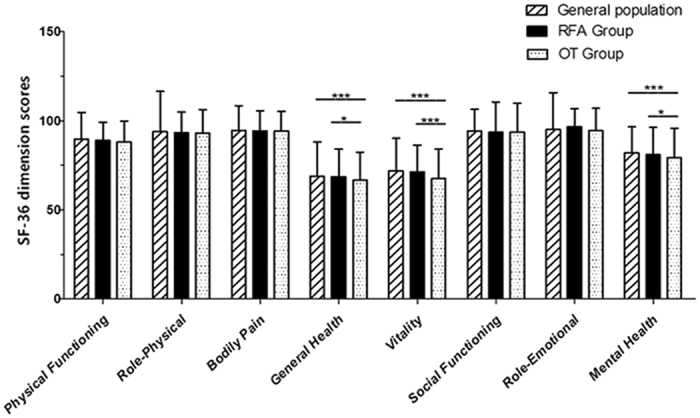
Graph shows the SF-36 dimension scores of the propensity score matched patients treated with radiofrequency ablation (RFA) or open thyroidectomy (OT) at 6 months follow up, together with those for the general population sample^13^. *P < 0.05,***P < 0.001.

The SF-36 scale scores of the propensity score mathed patients with BTNs that treated with RFA or OT at 6 months follow up are shown in Fig. 3, together with those for the general population sample^13^. 6 months after treatments, in the RFA group the HRQoL scores were significantly improved on GH (68.5 versus 65.3, *P* = 0.012), RE (96.6 versus 91.7, *P* = 0.007) and MH (80.9 versus 75.3, *P* = 0.002), and in the OT group they were improved on RE (94.4 versus 91.0, *P* = 0.049) and MH (79.3 versus 76.0, *P* = 0.011). Further, patients treated with RFA had significantly better HRQoL than patients treated with OT on GH (68.5 versus 66.7, *P* = 0.029), VT (71.3 versus 67.5, *P* < 0.001) and MH (80.9 versus 79.3, *P* = 0.038) at 6 months follow up (Fig. 3). And, compared the general population sample, the OT patients still exhibited significantly lower scores on three scales (GH: 66.7 versus 68.8, *P* < 0.001; VT, 67.5 versus 71.8, *P* < 0.001 and MH, 79.3 versus 81.8, *P* < 0.001), while there were no differences between the RFA group patients and the general population on all the scale scores at 6 months follow-up.

Using the EQ-5D-3L questionnaire, at baseline the OT patients had significantly more problems with usual activities and anxiety/depression than RFA patients (Table 3), translating into a lower utility score (estimated at 0.830 and 0.826, respectively). At 6 months after the treatment, mean QALYs reached 0.425 and 0.415 per patient treated by RFA or OT, respectively (Table 4). Because there were no between group differences in survival, the QALY differences were only explained by the utility values.

### Resource Use and Costs

Mean direct costs were estimated at Y18,209 (US$2,740) per RFA patient and Y12,439 (US$1,872) per OT patient. The use of RFA was associated with an extra cost of Y5,770 (US$868) per patient, mainly as a result of the high purchase price of the RFA device (Y13,600). The mean length of hospital stay was significantly shorter for RFA patients than OT patients (2.6 days versus 5.3 days, *P* < 0.001), leading to a lower indirect cost of the RFA group than the OT group (Y451 versus Y746, *P* < 0.001). However, the mean total cost of the RFA group was higher than that of the OT group (Y18,660 versus Y13,185, *P* < 0.001).

### Cost-Effectiveness Analysis

**Figure 4:**
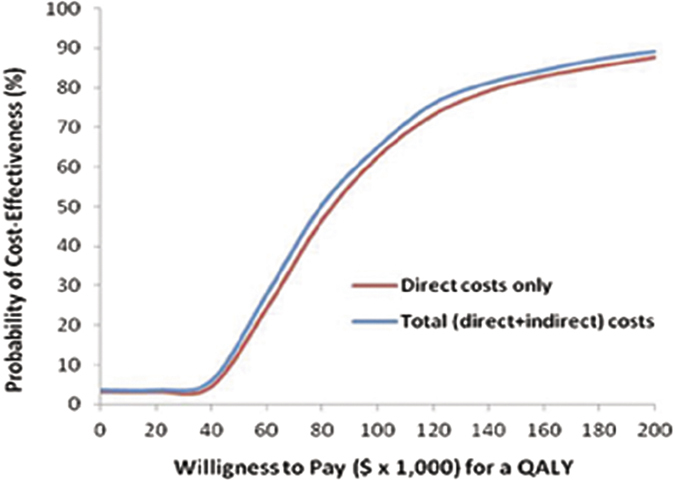
Acceptability curves of radiofrequency ablation (RFA) compared with open thyroidectomy (OT). Cost effectiveness acceptability curve using the net–monetary benefit approach (10,000 bootstrap replications) represents the probability (y-axis) that RFA is more cost effective compared with OT at the range of willingness-to-pay thresholds (US$ per quality-adjusted life-year [QALY]) on the x-axis. The curve is generated by repeating the procedure for various thresholds, with the threshold on x-axis and the probability of RFA to be cost effective on y-axis. Acceptability curves are presented here taking into account direct costs only or total (direct and indirect) costs.

Over the 6-month period, the use of RFA was more effective in terms of QALYs (mean increase of 0.01 QALY per patient) but was more expensive than OT (Table 4). With the threshold of US$50,000/QALY, the probability that RFA would be cost effective was 12.9% and 15.5% when direct and total (direct costs plus indirect costs) costs were considered, respectively (Fig. 4). When the RFA price was lowered by 10% or 30%, the extra cost incurred with the treatment procedure of RFA was reduced (Y1,360 versus Y4,080 for direct costs) and the probability that RFA would be cost effective for at the threshold of US$50,000/QALY increased to 36.6% and 88.4%, respectively (Supplemental. Fig. A1).

## Discussion

To our knowledge, this is the first study to compare between RFA and OT in the management of BTNs on parameters of QoL and cost-effectiveness. Our data showed that although HRQoL of the patients obtained a significant improvement after RFA or OT treatment, the HRQoL deficits persisted for the OT patients at 6 months follow up, compared to the general population. Also, HRQoL was better in three of the SF-36 domains for RFA patients than OT at 6 months follow up. Given the current price of RFA in the China context, it is unlikely to be cost effective at a threshold of US$50,000/QALY. The availability of an innovative RFA in the future might probably allow a price reduction and better value for money.

Nowadays, treatments of BNTs are primarily elected for the HRQoL indications, and therefore, well-controlled studies should be performed to compare the HRQoL effects of various treatment modalities^24^. Indeed, there are an increasing number of studies using HRQoL as a high priority outcome in the field of thyroid diseases and have reported heterogeneous results. Valcavi R *et al*.^25^ suggest that RFA can acquire an improvement of HRQoL in their 2-year follow-up study in 40 patients with BNTs, using the SF-36 questionnaire. However, applying the same questionnaire, Per Cramon P *et al*.^12^ carried out their study in patients with benign thyroid disease have HRQoL as the primary outcome demonstrated that HRQoL deficits persisted 6 months after standard treatments including radioactive iodine, hemithyroidectomy, total thyroidectomy and ethanol sclerotherapy. Differences in HRQoL results may be explained by the differences in sample sizes and inclusion criteria. Also, these previous studies are somewhat limited by the small sample sizes or inconsistent treatment modalities, and more important, regarding post-treatment HRQoL of patients with BNTs, no comparative study of RFA versus OT is available yet. In our study, the results showed that HRQoL of patients were significantly improved after RFA or OT treatment. Also, RFA patients had better HRQoL at follow-up compared with OT patients. Further, the absolute differences in scale scores are rather small, which means that even though they are significant the differences may not be clinically relevant (exceed the MID-minimal important difference-for the SF-36). So the advantage of RFA is: less invasive, lower rate of complications, no scar, shorter hospitalization but the observed differences in HRQoL may be too small too be clinical relevant Moreover, our data suggest that patients suffered HRQoL deficits 6 months after OT, compared to the general population. This was unexpected, and considering 8.6% OT patients suffered hypothyroidism, whether this result is directly associated with therapy or whether the impairments are caused by other factors remains unknown. However, this finding highlighted an important psychosocial factor to consider when giving medical care to patients with BTNs.

No previous studies have evaluated the cost-effectiveness of RFA for BTNs treatment, and the study is not a prospective cost-effectiveness analysis, but an approximation based on perceived costs related to medical care in these patients. In particular, the data might be sensitive to the sick leave or the RFA price. To account for some of that variability, a sensitivity analysis was performed in this study. Using a 6-month time horizon and a threshold of US$50,000/QALY, our results showed RFA to be a treatment modality with low probability of cost-effectiveness and was sensitive to the RFA price. 2015 American Thyroid Association guidelines^1^ did not include RFA for the treatment of patients with BTNs as a Class I recommendation. This may be attribute to the lack of mature RFA regulations, the probability of additional treatments, as well as to the expensive RFA procedures. The techniques of RFA and advanced ultrasonic medical devices used to facilitate RFA are ever evolving as we are learning more about this condition. The costs associated with RFA device may diminish over time as it penetrates into the mainstream.

Our study has several strengths. First, this study showed the first comparison result between RFA and OT on parameters of HRQoL and cost-effectiveness constitutes available evidence for guiding future clinical practice. Second, although this study was conducted as a retrospective analysis, an effectively and well calibrated propensity score model was used to compare HRQoL of two group patients. Third, the time horizon (6 months) used in this study was well fixed. On one hand, the healing of thyroid incision has entered its mature stage 6 months after hemithyroidectom, on the other hand nodule volume will gain a significant decrease 6 months after RFA^5^,^6^,^7^. Therefore, the clinical results of this study at 6-month visit were stable and meaningful.

Our study also has limitations. First, the 6-month follow-up in this study did not assess the longer-term effect on HRQoL of patients received medical treatment for BTNs and difference regarding incomplete data for the two groups (n = 81 vs. n = 2) was a little large. All RFA procedures were conducted by our radiologist, and meanwhile this study was led by radiologists but not the surgeons, and that might win a better compliance of the RFA patients than the OT patients. We think that is a main reason why there is a large difference regarding incomplete data for the two groups. Wickwar S *et al*.^26^ reported that the appearance-related social anxiety and avoidance caused by surgery were all found to improve as time goes on, suggesting that the HRQoL of patients take some time to change after OT. Thus long-term effect on patients of the two medical treatment should be verified. Second, the questionnaire used in this study was not directed against thyroid-specific quality of life, so the specificity of our study findings may be relatively poor. For example, the EQ-5D-3L questionnaire was found not to be quite sensitive in the oncology setting, especially in situations where level of vitality is an important element^27^. In the context of thyroid nodules, where vitality/fatigue is the most impacted domain by hypothyroidism, the EQ-5D-3Ltool might not capture small difference in QALYs between RFA and OT. A validated thyroid-specific survey^28^ would be expected to be more sensitive and responsive to changes in HRQoL than the generic survey (SF-36) used to assess HRQoL. Third, some data of this study may be specific to the China context. The cost of RFA was based on Shanghai Pricing Bureau for Public Third-senior Hospital. Also to explain the impact of the length of hospital stay on cost, sick leave was calculated based from China sick leave compensation system. However, the sick leave cost were relatively small, so its impact on cost-effectiveness is expected to be low.

In conclusion, this current retrospective cohort study demonstrated that both RFA and OT can obtain varying degrees of improvement in HRQoL of patients with BNTs. Although, compared with OT, RFA can better improve the post-treatment HRQoL, at its current price in the China context, it is unlikely to be cost-effective in short time. And RFA would be cost effective if price of the RFA device was lowered by 30%.

## Additional Information

**How to cite this article**: Yue, W.-W. *et al*. Quality of Life and Cost-Effectiveness of Radiofrequency Ablation versus Open Surgery for Benign Thyroid Nodules: a retrospective cohort study. *Sci. Rep.* **6**, 37838; doi: 10.1038/srep37838 (2016).

**Publisher's note:** Springer Nature remains neutral with regard to jurisdictional claims in published maps and institutional affiliations.

## Supplementary Material

Supplementary Information

## Figures and Tables

**Table 1:** Unit Cost and Sources.

Resource	RFA group	Unit Cost (Y)	OT group	Unit Cost (Y)	Source
Medical nursing	Second grade nursing cost (per day)	20	Special nursing cost (per day)	60	Shanghai Pricing Bureau for Public Third-senior Hospital, May, 2016
			First grade nursing cost (per day)	26	
			Second grade nursing cost (per day)	20	
Hospital Day	24 hours hospital stay	40	24 hours hospital stay	40	
	<24 hours hospital stay	20	<24 hours hospital stay	20	
Doctor visit	Radiological visit	10	Surgical visit	10	
Medical treatment procedure	RFA	2200	Thyroidectomy	2093	
	Thyroid biopsy	483	General anesthesia	800	
	Local infiltration anesthesia	8	Nerve block anesthesia	150	
			Postanesthesia Nursing	280	
Examination and laboratory test expenses	Electronic laryngoscope	150	Electronic laryngoscope	150	
	Ultrasound guidance	30	Intraoperative Ultrasound guidance	40	
	ECG	20	ECG	20	
	Chest X-ray	70	Chest X-ray	70	
	CEUS	200	Pathological examination	200	
	Thyroid function tests	362	Thyroid function tests	362	
	Calcium	5	Calcium	5	
	Coagulation function test (APTT, TT, D-Dimer, PT, plasma fibrinogen)	125	Coagulation function test (APTT, TT, D-Dimer, PT, plasma fibrinogen)	125	
	Anti-HIV	60	Anti-HIV	60	
	Anti-HCV	100	Anti-HCV	100	
	Treponema pallidum specific antibody	60	Treponema pallidum specific antibody	60	
	Hepatitis markers (HBsAg, HBsAb, HBeAg, HBeAb, HBcAb)	130	Hepatitis markers (HBsAg, HBsAb, HBeAg, HBeAb, HBcAb)	130	
	Complete blood count test	20	Hepatic and renal functions tests	93	
			Complete blood count test	20	
Drug	Levothyroxine (50 ng)	0.31	Propofol Injcetion (500 mg)	237	
	Lidocaine hydrochloride (5 ml)	0.4	Ropivacaine hydrochloride (100 mg)	31.9	
			Sevoflurance (1 ml)	8.03	
			Dexmedetomidine Hydrochloride Injection (200 ug)	186	
			Hemocoagulase Injection (1ku)	41.5	
			Cisatracurium Besilate for Injection (10 mg)	101	
			Remifentanil (1 mg)	104	
			Levothyroxine (50 ng)	0.31	
			Penehyclidine Hydrochloride (1 mg)	53.5	
Instruments and Materials	PTC puncture needle	95	Ultrasonic scalpel	1400	
	RFA instrument	13600	Highfrequency electrotome	150	
			Peripheral nerve stimulator	240	
			Steel wire tube	210	
			Negative pressure drainage bottle	278	
			Medical adhesive (1.5 ml)	560	
			SURG TAKE (250 ml)	490	
			Disposable tracheal intubation instrument	70	
Indirect costs	Value of GDP Per Capita (per day)	267	Value of GDP Per Capita (per day)	267		National Bureau of Statistic for Shanghai, China, December, 2014

Note: Y1 = US$0.1505; RFA group, patients treated with radiofrequency ablation; OT group, patients treated with open thyroidectomy; ECG: electrocardiogram; CEUS: contrast-enhanced ultrasound; APTT: activated partial thromboplastin time; TT: thrombin time; D-Dimer, PT: thromboplastin time; GDP: Gross Domestic Product.

**Table 2:** Baseline characteristics of patients with benign thyroid nodules treated with radiofrequency ablation and open thyroidectomy before and after Propensity Score Matching.

Characteristics	Before matching			After matching		
	RFA n = 137	OT n = 267	*P* value	RFA n = 108	OT n = 108	*P* value
Age (y)	48.3 ± 12.9	52.4 ± 12.7	0.003	50.8 ± 11.9	49.8 ± 13.6	0.532
Sex			<0.001			0.775
Male	38(27.7)	129(48.3)		36(33.3)	39(36.1)	
Female	99(72.3)	138(51.7)		72(66.7)	69(63.9)	
Ethnic group			0.862			0.701
Han-Chinese	133(97.1)	260(97.4)		105(97.2)	104(96.3)	
Other	4(2.9)	7(2.6)		3(2.8)	4(3.7)	
SF-36 dimension scores						
PF	87.8 ± 11.2	86.9 ± 13.8	0.510	87.5 ± 10.8	87.1 ± 13.2	0.778
RP	91.8 ± 12.9	91.2 ± 10.6	0.690	91.7 ± 12.8	90.3 ± 10.2	0.469
BP	92.2 ± 13.2	94.4 ± 10.9	0.085	92.9 ± 12.8	94.1 ± 11.1	0.480
GH	65.8 ± 15.5	64.5 ± 16.4	0.036	65.3 ± 15.6	64.9 ± 16.2	0.647
VT	69.2 ± 15.2	69.1 ± 17.4	0.913	69.4 ± 15.3	69.1 ± 17.4	0.792
SF	93.2 ± 17.6	91.9 ± 17.4	0.102	92.5 ± 17.8	92.5 ± 16.7	0.945
RE	92.7 ± 14.9	88.9 ± 18.2	0.035	91.7 ± 15.8	91.0 ± 15.5	0.773
MH	75.7 ± 16.1	74.1 ± 16.8	0.017	75.3 ± 16.3	76.0 ± 15.7	0.394
Smoke			0.929			1.000
Yes	37(27)	71(26.6)		28(25.9)	28(25.9)	
No	100(73)	196(73.4)		80(74.1)	80(74.1)	
Drink			0.890			0.785
Yes	57(41.6)	113(42.3)		48(44.4)	50(46.3)	
No	80(58.4)	154(57.7)		60(55.6)	58(53.7)	
Chronic diseases			0.976			0.884
Yes	47(34.3)	92(34.5)		35(32.4)	34(31.5)	
No	90(65.7)	175(65.5)		73(67.6)	74(68.5)	
Marital status			0.197			0.984
Unmarried	12(8.8)	15(5.6)		7(6.5)	8(7.4)	
Married	118(86.1)	224(83.9)		94(87)	94(87)	
Widowed	6(4.4)	26(9.7)		6(5.6)	5(4.6)	
Divorced	1(0.7)	2(0.7)		1(0.9)	1(0.9)	
Other	0(0)	0(0)		0(0)	0(0)	
Monthly income (Y)			0.337			0.757
<3000	23(16.8)	36(13.5)		14(13)	13(12)	
3000–7999	69(50.4)	152(56.9)		55(50.9)	58(53.7)	
>8000	34(24.8)	67(25.1)		29(26.9)	31(28.7)	
data privacy	11(8)	12(4.5)		10(9.3)	6(5.6)	
Education lever			0.147			0.441
Primary education and lower	9(6.6)	29(10.9)		9(8.3)	9(8.3)	
Secondary/high education	62(45.3)	133(49.8)		56(51.9)	47(43.5)	
University education and higher	66(48.2)	105(39.3)		43(39.8)	52(48.1)	
Current job			0.004			0.900
Manual worker	20(14.6)	17(6.4)		11(10.2)	11(10.2)	
Office worker	55(40.1)	85(31.8)		39(36.1)	44(40.7)	
Retire	50(36.5)	128(47.9)		46(42.6)	43(39.8)	
Other	12(8.8)	37(13.9)		12(11.1)	10(9.3)	
Nodule volume (ml)^a^	5.7(3.9–9.1)	5.2(1.4–11.2)	0.026	5.6(3.9–8.7)	5.3(2.2–10.4)	0.104

Note: RFA group, patients treated with radiofrequency ablation; OT group, patients treated with open thyroidectomy; Except for nodule volume and P values, data are reported as No. (%) or mean ± standard deviations.

^a^Data are with skewed distribution and are reported as median with the inter-quartile range in parentheses and analyzed using Mann-Whitney U test; SF-36, Short Form-36, PF = Physical Functioning, RP = Role-Physical, BP = Bodily Pain, GH = General Health, VT = Vitality, SF = Social Functioning, RE = Role-Emotional, MH = Mental Health.

**Table 3:** Descriptive results obtained with the EuroQoL-5D-3L at baseline by methods of radiofrequency ablation and open thyroidectomy.

Dimension and Level	RFA n = 137	OT n = 267	*P* value
Mobility			0.09
No problems (1)	108(78.8)	229(85.5)	
Some problems (2)	29(21.2)	38(14.2)	
Confined to bed (3)	0(0)	0(0)	
Self-care			0.095
No problems (1)	131(95.6)	263(98.5)	
Some problems (2)	6 (4.4)	4(1.5)	
Unable to (3)	0(0)	0(0)	
Usual activities			0.042
No problems (1)	110(80.3)	188(70.4)	
Some problems (2)	27(19.7)	79(29.6)	
Extreme (3)	0(0)	0(0)	
Pain discomfort			0.054
None (1)	86(62.8)	193(72.3)	
Moderate (2)	51(37.2)	74(27.7)	
Extreme (3)	0(0)	0(0)	
Anxiety/depression			0.004
None (1)	85(62)	119(44.6)	
Moderate (2)	50(36.5)	144(53.9)	
Extreme (3)	2(1.5)	4(1.5)	
Patients in perfect health state (all levels = 1)	40(29.2)	61(22.8)	
Utility score	0.830 ± 0.093	0.826 ± 0.085	

Note: RFA group, patients treated with radiofrequency ablation; OT group, patients treated with open thyroidectomy. Except for Utility score and *P* values, data are reported as No. (%).

**Table 4:** QALYS and Costs of patients with benign thyroid nodules treated with radiofrequency ablation and open thyroidectomy.

QALY and Cost	RFA Group	OT Group
QALYs	0.425 (0.418 to 0.432)	0.415 (0.411 to 0.420)
Direct cost (Y)	18209 (18136 to 18275)	12439 (12370 to 12505)
Indirect cost (Y)	451 (512 to 484)	746 (681 to 819)
Total cost (direct + indirect) (Y)	18660 (18577 to 19739)	13185 (13081 to 13281)

Note: QALY, quality adjusted life year. RFA group, patients treated with radiofrequency ablation; OT group, patients treated with open thyroidectomy; Data are reported as mean (95% CI); The 95% CIs are based on the bootstrap analyses; Costs are expressed in :Y1 = US$0.1505.
