# Corrigendum: Quality of Life and Cost-Effectiveness of Radiofrequency Ablation versus Open Surgery for Benign Thyroid Nodules: a retrospective cohort study

**DOI:** 10.1038/srep41342

**Published:** 2017-02-10

**Authors:** Wen-Wen Yue, Shu-Rong Wang, Feng Lu, Xiao-Long Li, Hui-Xiong Xu, Li-Ping Sun, Le-Hang Guo, Ya-Ping He, Dan Wang, Zhi-Qiang Yin

Scientific Reports 6: Article number: 37838; 10.1038/srep37838 published online: 11 24 2016; updated: 02 10 2017.

Shu-Rong Wang was omitted from the author list in the original version of this Article. This has been corrected in the PDF and HTML versions of the Article and in the supplementary information file.

Acknowledgements

“Supported in part by the Shanghai Hospital Development Center (Grant SHDC 12014229), the Science and Technology Commission of Shanghai Municipality (Grants 14441900900 and 16411971100), and the National Natural Scientific Foundation of China (Grant 81601502)”.

Now reads:

“Supported in part by the Shanghai Hospital Development Center (Grant SHDC 12014229), the Science and Technology Commission of Shanghai Municipality (Grants 14441900900 and 16411971100), and the National Natural Scientific Foundation of China (Grant 81601502). Thank Prof Shu-Rong Wang for data collection”.

Contributions

“Concept and design: H.X. and W.Y. Performance of RFA and OT: F.L. and Z.Y. Collection and assemble of data: W.Y., L.S. and X.L. Data analysis and interpretation: W.Y., Y.H., D.W. and L.G. Manuscript written: W.Y. Reviewed the manuscript: All authors”.

Now reads:

“Concept and design: H.X., S.W. and W.Y. Performance of RFA and OT: S.W., F.L and Z.Y. Collection and assemble of data: W.Y., L.S., S.W.and X.L. Data analysis and interpretation: W.Y., Y.H., D.W. and L.G. Manuscript written: W.Y. Reviewed the manuscript: All authors”.

