# Supplementary material for: Quality of Life and Cost-Effectiveness of Radiofrequency Ablation versus Open Surgery for Benign Thyroid Nodules: a retrospective cohort study
Source: Sci Rep. 2016 Nov 24;6:37838. doi: 10.1038/srep37838 (PMC5121639; doi:10.1038/srep37838)
Supplement: Supplementary Information [file srep37838-s1.pdf]

Quality of Life and Cost-Effectiveness of Radiofrequency Ablation versus Open Surgery for Benign Thyroid Nodules: a retrospective cohort study

Wen-Wen Yue[1,2,3], Xiao-Long Li[1,2,3], Hui-Xiong Xu[1,2,3*], Feng Lu[1,2,3], Li-Ping Sun[1,2,3], Le-Hang Guo[1,2,3], Ya-Ping He[1,2,3], Dan Wang[1,2,3] and Zhi-Qiang Yin [2,3,4]

*Correspondence: Hui-Xiong Xu, Department of Medical Ultrasound, Shanghai Tenth People's Hospital, Ultrasound Research and Education Institute, Tongji University School of Medicine, No. 301, Yanchangzhong Road, Shanghai 200072, China (E-mail: xuhuixiong@126.com).

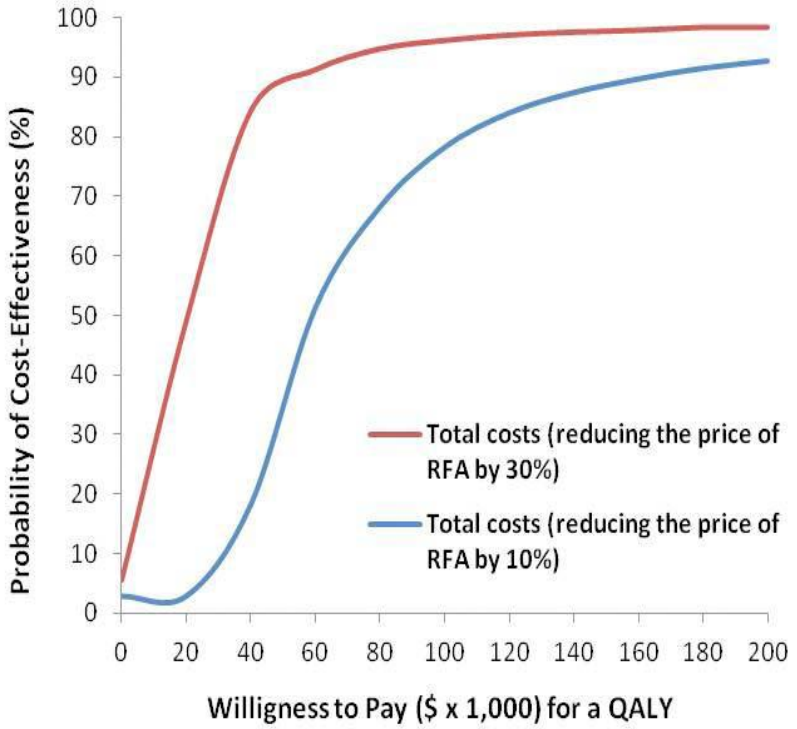

Supplemental. Figure-A1. Acceptability curves of radiofrequency ablation (RFA) compared with open thyroidectomy (OT). Cost-effectiveness acceptability curve using the net–monetary benefit approach (10,000 bootstrap replications) represents the probability (y-axis) that RFA is more cost-effective compared with OT at the range of willingness-to-pay thresholds (US$ per quality-adjusted life-year [QALY]) on the x-axis. The curve is generated by repeating the procedure for various thresholds, with the threshold on x-axis and the probability of RFA to be cost-effective on y-axis. Acceptability curves are presented here taking into account total costs (reducing the price of RFA by 30%) or total costs (reducing the price of RFA by 10%).
